# Biodistribution and toxicity assessment of methoxyphenyl phosphonium carbosilane dendrimers in 2D and 3D cell cultures of human cancer cells and zebrafish embryos

**DOI:** 10.1038/s41598-023-42850-3

**Published:** 2023-09-19

**Authors:** Zuzana Žmudová, Zuzana Šanderová, Michaela Liegertová, Stanislav Vinopal, Regina Herma, Luděk Sušický, Monika Müllerová, Tomáš Strašák, Jan Malý

**Affiliations:** 1 CENAB, Faculty of Science, Jan Evangelista Purkyně University in Ústí Nad Labem, Ústí nad Labem, Czech Republic; 2 Institute of Chemical Process Fundamentals of the CAS, Prague, Czech Republic

**Keywords:** Biological techniques, Biotechnology, Cell biology, Molecular biology, Natural hazards, Risk factors, Nanoscience and technology

## Abstract

The consideration of human and environmental exposure to dendrimers, including cytotoxicity, acute toxicity, and cell and tissue accumulation, is essential due to their significant potential for various biomedical applications. This study aimed to evaluate the biodistribution and toxicity of a novel methoxyphenyl phosphonium carbosilane dendrimer, a potential mitochondria-targeting vector for cancer therapeutics, in 2D and 3D cancer cell cultures and zebrafish embryos. We assessed its cytotoxicity (via MTT, ATP, and Spheroid growth inhibition assays) and cellular biodistribution. The dendrimer cytotoxicity was higher in cancer cells, likely due to its specific targeting to the mitochondrial compartment. In vivo studies using zebrafish demonstrated dendrimer distribution within the vascular and gastrointestinal systems, indicating a biodistribution profile that may be beneficial for systemic therapeutic delivery strategies. The methoxyphenyl phosphonium carbosilane dendrimer shows promise for applications in cancer cell delivery, but additional studies are required to confirm these findings using alternative labelling methods and more physiologically relevant models. Our results contribute to the growing body of evidence supporting the potential of carbosilane dendrimers as vectors for cancer therapeutics.

## Introduction

Nanoparticles, such as polymeric nanoparticles^1^, liposomes^2^, polymer micelles^3^, and virus-based nanoparticles^4,^ are becoming increasingly popular in biotechnology and biomedicine. Dendrimers are monodisperse polymeric structures possessing a regular, highly branched three-dimensional architecture, whose molecular weight and shape can be easily and precisely predicted by computer modelling^1^. These nanoparticles are typically composed of a central core molecule surrounded by dendrons, which may be terminated with various functional peripheral groups^5^. Dendrimers have been utilised in a variety of fields, including catalysis, sensors, surface engineering, biomedical science, or photochemistry^6^. In biomedicine, dendrimers can be used as vector systems for targeted drug delivery and gene therapy^7^. Dendrimers with a positive surface charge, such as polyamidoamine (PAMAM) and polypropyleneimine (PPI), can bind and protect nucleic acids prior to transfection (dendritic encapsulation)^8^, or they can be used to target drugs into specific organelles, i.e., mitochondria, to facilitate pharmacological action^9^.

With such enormous potential for application, human and environmental exposure to dendrimers (acute toxicity and ecotoxicity, as well as cell and tissue accumulation) must be considered. However, studies examining the potential risk associated with nanoparticle exposure are still limited. Most dendrimer toxicity studies were performed in standard in vitro conditions^10^, with only a few studies focused on the whole organism level (acute and developmental toxicity)^11^. Dendrimers terminated with neutral or anionic groups (e.g. carboxylic acid or hydroxyl-terminated polyamidoamines) generally show lower toxicity^12^ when compared to cationic dendrimers (e.g., NH_2_-terminated PAMAM and polypropylenimine dendrimers), where the positive surface charge leads to strong interaction with anionic lipid bilayers (biological membranes), causing the formation of nanopores, eventually leading to cell death^13^. Surface modification, for example, of the most common PAMAM dendrimers^14^, or the synthesis of supposedly more biocompatible variants such as phosphorus^15^, polyester^16^, triazine^17^, or carbosilane^18^ dendrimers can reduce overall toxicity. Of note, there are only a few studies addressing the toxicity of these novel types of dendrimers^15,19,20^, and the comparisons of in vitro and in vivo toxicity or cellular and tissue biodistribution are largely lacking.

The methoxyphenyl phosphonium dendrimer (PhO) emerged as a compound of interest in our previous research^20,21^, exhibiting attributes that highlight its potential as a promising mitochondria-targeting vector in cancer cell delivery of therapeutics. A series of experiments conducted across diverse non-cancer mammalian cell lines revealed its relatively low cytotoxicity, a feature that was consistently observed in both 3-(4,5-dimethylthiazol-2-yl)-2,5-diphenyl-2H-tetrazolium bromide (MTT) and Crystal Violet (CV) assays^20^. Remarkably, the cytotoxic profile of this dendrimer paralleled those of Poly(ethylene glycol) (PEG) and sugar-coated dendrimers^19^, underscoring its promising biocompatibility and its potential role in biomedical applications. Our prior investigations led us to propose a mechanism that attributes the low cytotoxicity of the PhO dendrimer to a specific electronic configuration facilitated by its unique structure. The presence of three electron-donating methoxy groups in para positions appears to instigate a delocalization of the positive charge on the phosphorus atoms, thereby mitigating potentially toxic interactions with cellular structures such as the cell membrane and intracellular proteins^20,21^. Additionally, PhO dendrimer displayed an enhanced water solubility of more than 100 mg/mL. This solubility, achieved through the strategic exchange of iodide anions for chloride, is critical for any prospective therapeutic agent, influencing its absorption, distribution, metabolism, and excretion^20^.

To explore its potential further, we aimed to examine its activity in a model human cancer cell line and in an in vivo model organism, *Danio rerio*. The MCF-7 breast cancer cell line was chosen as the model for this study due to its widespread use and relevance in cancer research^22^. Their ease of culture, high proliferation rate, well-characterised behaviour, and suitability for 3D cancer cell spheroids cultivation^23^ make them a valuable tool for in vitro studies.

Cells in tumour spheroids grow and interact in all three dimensions, which is regarded as a more reliable arrangement than in traditional 2D cell cultures^24^. This system better reflects the organism’s natural environment in terms of cell–cell interactions^25^, nutrient and oxygen gradients^26^ and could be used to assess cumulative effects and cellular biodistribution of dendrimers^27^. We used this in vitro 3D model system to complement the results from the monolayer cell cultures.

The zebrafish (*Danio rerio*) is a well-established, time- and cost-effective vertebrate model system with high genetic homology to mammals^28^, for studying toxicity and biodistribution in vivo. The fish embryo test (FET) for assessing acute and developmental toxicity is standardised by the OECD^29^, widely accepted, and has been shown to have a high correlation with results in humans^30^.

In this study, we systematically analysed the cellular toxicity and intercellular as well as systemic accumulation patterns of PhO dendrimer in in vitro and in vivo contexts. The in vitro cytotoxicity experiments were performed using a model MCF-7 breast cancer cell line in the form of a classical 2D monolayer (ATP assay, MTT assay) and in the form of 3D spheroids (ATP assay, Spheroid growth inhibition assay). We performed a spheroid biodistribution assay to gain physiologically relevant insights into the accumulation of dendrimers within cancer cells. Finally, our study aimed at evaluating dendrimer acute toxicity and accumulation patterns at the whole-organism level in zebrafish embryos. This sequential methodology, moving from a simplistic to a more complex biological model, provides a robust evaluation of PhO dendrimers with respect to their potential application in cancer cell-delivery therapies.

## Materials and methods

### Dendrimers synthesis and preparation

The third-generation cationic carbosilane dendrimer used in this work was functionalized with the methoxyphenyl-phosphonium -P(C_6_H_4_-OMe)_3_ peripheral substituents. The preparation protocols, structural features, and thorough characterization of these dendrimers were previously described in detail elsewhere^20,21^. For the experiments, the dried PhO powder was directly mixed into cell culture media and zebrafish embryo water, taking advantage of its excellent solubility. Stock solutions were prepared fresh before each experiment and immediately diluted to achieve the desired concentrations for each experimental setup.

### 2D in vitro toxicity assays

#### Cell cultivation

The human breast cancer cell line MCF-7 (Homo sapiens, ATCC, HTB-22, Sigma-Aldrich) was maintained in Dulbecco’s modified Eagle’s medium (D-MEM) supplemented with 10% (v/v) foetal bovine serum, 0.1% (w/v) penicillin, 0.1% (w/v) streptomycin, and 0.25% (w/v) amphotericin. Cells were routinely maintained on plastic tissue culture dishes (Greiner) at 37 °C in a humidified atmosphere of 5% CO_2_/95% air (ESCO incubator). Cells were harvested at 80–90% confluence and used in experiments. The number of viable cells was determined by trypan blue exclusion on a hemocytometer.

#### MTT assay

Cells were seeded (10 000 cells/well) in 96-well plates (Thermo Fisher Scientific) and grown in an appropriate growth medium for 24 h. The cells were then treated with PhO at concentrations ranging from 0.05 to 5 µM. After 24 h of incubation, 50 µl of a solution of MTT (5 mg/10 mL) in sterile PBS was added to each well. Four hours later, the MTT was removed, the formazan precipitate was dissolved in DMSO and the absorbance was measured at 580 nm and reference 700 nm. Viability is graphically presented as a percentage of the control values (without dendrimers). The IC_50_ value was acquired through nonlinear regression analysis (GraphPad Software, Version 9).

#### ATP assay

Cells were seeded (10 000 cells/well) into 96-well plates and grown for 24 h in 100 µL of appropriate growth medium. Cells were then treated with PhO concentrations ranging from 0.05 to 10 µmol/L. After 24, 48, 72, and 96 h of incubation, the plate and its contents were equilibrated at room temperature for approximately 30 min. A volume of CellTiter-Glo reagent equal to the volume of cell culture medium present in each well was then added, and the contents were mixed for 5 min to induce cell lysis. The plate was then incubated at room temperature for 15 min to stabilise the luminescence signal. Luminescence was recorded using a GloMax Explorer Multimode Microplate Reader (Promega). The proliferative response of the treated cells was normalised to the average amount of ATP at zero dendrimer concentration and plotted in a dose- and time-dependent manner. IC_50_ values were acquired through nonlinear regression analysis (GraphPad Software, Version 9).

### 3D in vitro toxicity assays

#### Cultivation and characterization of 3D spheroids

A 3D Petri dish (MicroTissues) was used to agglomerate MCF-7 cells into 3D spheroids. The 3D Petri dish was transferred into a 12-well culture plate (Thermo Fisher Scientific). For spheroid formation, a density of 1.2 × 10^6^ MCF-7 cells per 1 ml was used. After seeding cells in a 3D Petri dish, 2.5 ml of D-MEM (Gibco) culture medium was added. Plates with 3D Petri dishes containing cells were then incubated for 72 h at 37 °C with 5% CO_2_ to allow cells to form spheroids. Spheroid morphology was investigated for 96 h by an inverted microscope (Olympus IX73), using spheroids incubated with PhO at a concentration range of 0.05 to 10 µmol/L. Untreated spheroids were used as a negative control.

#### ATP spheroid assay

To avoid interference of the dendrimer with spheroid formation, spheroids were enabled to form before the PhO treatment. After the 72 h of incubation that were necessary for proper spheroid formation, the medium was removed, and the spheroids were exposed to the dendrimer dissolved in the fresh D-MEM medium. Spheroids were treated with the dendrimer at concentrations ranging from 0.05 to 10 µmol/L. The luminescence method using the CellTiter-Glo (G7571, Promega) assay was used to determine the cytotoxic effects of dendrimers according to the manufacturer’s protocol. 25 µL of spheroid medium (D-MEM) together with a single spheroid were carefully removed from each 3D Petri dish and transferred to an opaque-walled 96-well plate, and 25 µL of CellTiter-Glo reagent was added. ATP content in spheroids was measured in 24 h intervals for 96 h after PhO addition by a luminescence microplate reader (GloMax, Promega). Six spheroids were randomly selected to determine the cytotoxicity of PhO. Untreated spheroids were used as a negative control. The proliferative response of the treated cells was normalised to the average amount of ATP in the spheroids after 24 h at zero dendrimer concentration and plotted in a dose- and time-dependent manner. IC_50_ values were acquired through nonlinear regression analysis (GraphPad Software, Version 9).

#### Spheroid growth inhibition assay

The cytotoxicity effect of PhO was also evaluated by spheroid growth curves. After the 72 h necessary for spheroid formation, the medium was removed, and the spheroids were exposed to the dendrimer dissolved in the fresh D-MEM (similarly to the ATP assay). Seven spheroids for each concentration were selected for measuring the spheroids’ area. Spheroids were analysed and imaged by an inverted microscope (Olympus IX73) at 24-h intervals for 96 h after PhO addition. The area of spheroids was measured by CellSens software (Olympus). Untreated spheroids were used as a negative control.

### Biodistribution of PhO in 3D spheroids

3 × 10^5^ per 1 mL MCF-7 cells were seeded into a 24-well plate well with an agarose 3D Petri dish (MicroTissues) and incubated in the presence of D-MEM/10% FBS (media) including 1% B27, epidermal growth factor EGF (20 ng/mL), basic fibroblast growth factor bFGF (20 ng/mL) (all Gibco, ThermoFisher) for 48 h to allow the formation of compact 3D spheroids.

Next, the PhO dendrimer, labelled using fluorescent, photostable cyanine dye 5 (PhO-Cy5), was added into the 3D Petri dish media with spheroids to a final concentration of 1 μmol/L and incubated for 72 h. Untreated spheroids were used as a negative control.

The spheroids were harvested by gently inverting the 3D Petri dish into a 250 µL drop of prewarmed media placed on the lid of a sterile 6 cm culture dish and by subsequent gentle mechanical tapping on the 3D Petri dish. Free-floating spheroids were transferred using a cut pipette tip into an Eppendorf tube and washed three times with 0.5 mL of media. Spheroids were left to sediment by gravity between the washing steps. Washed spheroids were stained with a mixture of 10 μg/mL Hoechst 33342 and 100 nM Mitotracker Red (Thermo Scientific, M7512) diluted in media and incubated in the incubator for 45 min. Stained spheroids were washed four times with 0.5 mL of media. Washed spheroids were transferred into a µ-Slide 18-Well Glass Bottom (Ibidi, 81817) at a volume of 60 μL of media. A 15 µl drop of 2% low-melting agarose dissolved in OPTI-MEM was added to each sample to decrease the motion of spheroids inside the imaging chamber.

Spheroids were imaged using a Leica SP8 confocal microscope enclosed in an environmental chamber with constant temperature (36.9 °C), humidity (95%) and 5% CO_2_ levels. The Z-stacks of the whole spheroids were acquired using a 20x/0.75 N.A. dry objective with 2.5 μm intervals between slices. Intracellular details were imaged using a 63x/1.40 N.A. oil objective with a 1 μm interval between slices. Illumination intensity, filter, and detector settings were kept constant between control and PhO-Cy5-treated samples for a given objective. Filter and detector settings were optimised to minimise any possible bleed-through between fluorescence channels.

### In vivo toxicity

#### Fish maintenance

Adult zebrafish (wild-type and transparent Casper variants) were kept in constant light conditions (14 h of light, 10 h of dark photoperiod), with 20 individuals per aquarium (60 L). One day prior to the experiment, fish were transferred into spawning cages at the end of the photoperiod in a ratio of 3 males to 2 females. All fish spawned at the onset of the following photoperiod. Fertilised eggs were collected within 30 min post-fertilisation, inspected for their health state and developmental stage using a stereomicroscope, and either transferred to pre-prepared solutions of tested substances for incubation (FET) or reared in plastic Petri dishes into stages suitable for biodistribution assays.

#### Fish embryo test (FET)

Modified FET^29^ was performed on wildtype embryos according to the procedure described elsewhere^31^.All tested solutions were prepared with aerated E3 medium (5 mM NaCl; 0.17 mM KCl; 0.33 mM CaCl_2_; 0.33 mM MgSO_4_). For the FET, six different concentrations of the dendrimer were tested (100 µmol/L; 10 µmol/L; 1 µmol/L; 0.1 µmol/L; 0.01 µmol/L and 0.001 µmol/L). Each FET test consisted of 24 embryos per concentration, where each single embryo was considered an individual replicate. Fish embryos were inspected and scored for mortality (lethal endpoints) at four time points: 24, 48, 72, and 96 h post exposure. Six morphological endpoints were considered in our analysis: (**i**) coagulation, (**ii**) lack of somite formation, (**iii**) growth retardation, (**iv**) malformation, (**v**) lack of the tail bud from the yolk sac, and (**vi**) heart oedema.

### Biodistribution of PhO-Cy5 conjugate in zebrafish embryos

The distribution of PhO in zebrafish larval tissues was inspected using embryos of *Danio rerio* type Casper. The experiment was performed either by direct exposure to 1 µmol/L PhO-Cy5 or by injection of 50 µmol/L solutions into the embryos via the yolk sac. The first method included incubation of intact or dechorionated embryos with the PhO-Cy5 conjugate. Two hours post-fertilisation, the eggs were transferred into 24-well plates filled with 2 ml of 1 µmol/L PhO-Cy5. Half of the embryos were manually released from the chorion after 24 h of incubation. The well-plate was incubated at 28 °C, and the embryos were inspected by a fluorescent microscope every 24 h. All embryos were carefully rinsed with E3 medium^32^ before examination. Eight embryos were used as biological replicates for each treatment (8 intact, 8 dechorionated, and 8 negative controls).

The second method involved the direct injection of PhO-Cy5 into the yolk sac of 24-h-old embryos. Prior to injection, embryos were anaesthetized with Tricaine-3-aminobenzoic acid ethyl ester, also called ethyl 3-aminobenzoate (Sigma). The Tricaine anaesthetic solution was prepared by mixing 400 mg of tricaine powder dissolved in 97.9 mL of distilled water with 2.1 mL of 1 M Tris (pH 9), and the pH was adjusted to 7 (4.2 mL of this solution was diluted by 100 mL of E3 medium^33^). Anaesthetized embryos were immobilised in grooves (1 mm in diameter) formed on 1.5% agarose blocks submerged in E3 medium pre-warmed to 28 °C. Borosilicate glass capillaries were pulled to needles (inner diameter of 18 µm and an outer diameter of 20 µm^34^), and injections were carried out manually using the MPPI-3 Pressure Injector (Applied Scientific Instrumentation). The injection volume was calibrated to deliver 2.5 µL of solution per application. For the biodistribution assessment by injection, five individual embryos (biological replicates) were imaged and analysed. After each procedure, embryos were immediately transferred to fresh media to recover. Micrographs were taken with an inverted fluorescence microscope Olympus IX71. In accordance with established protocols, zebrafish embryos used in the experimental procedures were humanely euthanized by immersing them in a euthanasia bath containing 300 mg/L tricaine methanesulfonate anaesthetic solution. All experimental protocols were approved by the Institutional Committee for Animal Welfare; all methods were carried out in accordance with relevant guidelines and regulations; and the methods for working with zebrafish larvae are reported in accordance with ARRIVE guidelines where applicable.

### Statistical analysis

#### In vitro cytotoxicity assays

The data were displayed as a mean value ± standard deviation (SD). The spheroid growth was measured on seven randomly selected 3D spheroids based on their brightfield image area. The mean spheroid area at each timepoint was normalised by the mean spheroid area at 0 h (before the addition of PhO) for each condition. The growth curves were constructed by fitting the equation Y = Y/K, where K stands for negative control. The significance of differences was calculated using a two-way ANOVA followed by Dunnett’s post hoc test.

The inhibitory concentration value (IC_50_) and 95% confidence interval (CI) from MTT and ATP assays were derived from a nonlinear regression model based on a sigmoidal dose–response curve using nonlinear regression analysis. Dose–response curves were fitted using an equation: Y = Bottom + (Top–Bottom)/(1 + (IC_50_/X)^HillSlope).

The graphics and all statistical analyses were performed using GraphPad Prism (GraphPad Software, version 9).

#### FET data evaluation

LD_50_ value for the mortality recorded at 96 h, including the 95% CI, was determined. The percentage of embryos displaying lethal or sub-lethal morphological effects considered endpoints was plotted against the concentration scale. The data was analysed using probit analysis, and to estimate lethal concentrations, statistical analysis was conducted using a simple R-based function^35^ developed in the R code^36^.

### Ethics approval

We confirm that all the research meets ethical guidelines and adheres to the legal requirements of the study country. The University of Jan Evangelista Purkyně is a certified facility for the use of animals in research (Veterinary Approval Number CZ 42760032, Ministry of Agriculture of the Czech Republic Approval Number MZE-19331/2022-13143). The experimental project was approved by the Ministry of Education of the Czech Republic (Approval Number: MSMT-8474/2018-3) and by the Institutional Committee for Animal Welfare. ML, ZŠ, SV are certified for planning and performing experiments on animals.

## Results

### In vitro toxicity

**Figure 1:**
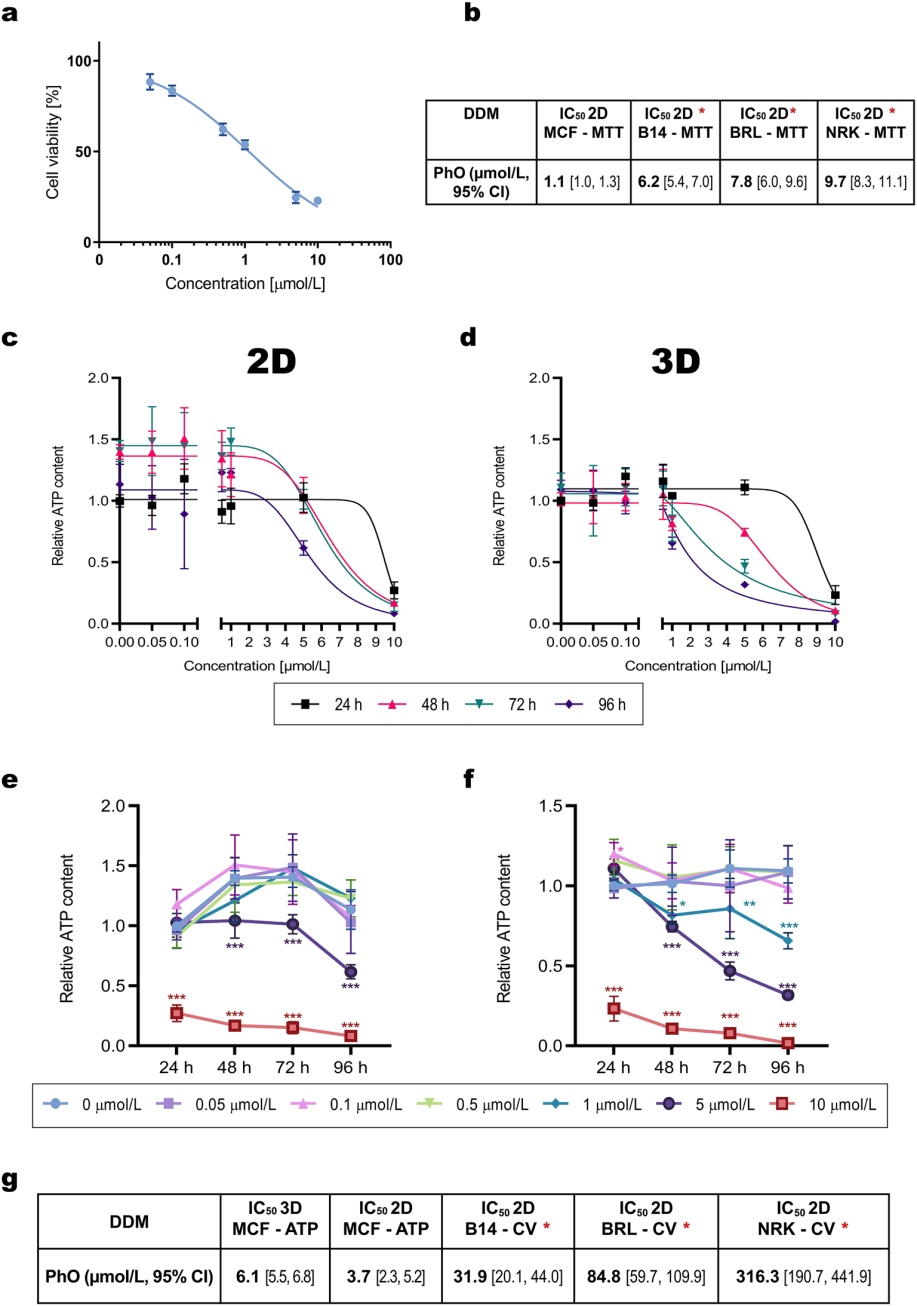
MTT and ATP Assays in 2D and 3D Cell Cultures. (**a**) Cytotoxicity of PhO to MCF-7 cells after 24-h exposure determined by the MTT assay. (**b**) Comparison of MTT assays with previously published results^20^. Cytotoxic effects of PhO using an ATP assay in 2D MCF-7 cell culture in a concentration- (**c**) and time- (**e**) dependent manner. Cytotoxic effects of PhO using an ATP assay in 3D MCF-7 spheroids in a concentration- (**d**) and time- (**f**) dependent manner. Statistical significance for (**e**, **f**): was determined using the two-way ANOVA followed by the Dunnett’s post hoc test. *p < 0.033, **p < 0.002, ***p < 0.001. (**g**) Comparison of cytotoxicity between the (ATP) ATP assay (this study) and previously published Crystal Violet (CV) assays. B14 (*Cricetulus griseus*), BRL (*Rattus norvegicus*), NRK (*Rattus norvegicus*) mammalian cell lines. Red asterisk indicates previous work by^20^. DDM—dendrimer.

The cytotoxicity of PhO was first examined using the classical 2D monolayer MCF-7 cell culture by the MTT assay and ATP assay. The MTT assay is a colorimetric assay based on the monitoring of NAD(P)H-dependent cellular oxidoreductase enzymatic activity and is therefore related to inhibition of metabolic processes in mitochondria^37^. The MTT-based IC_50_ value for MCF-7 cells was 1.1 µmol/L (95% CI [1.0, 1.3]; Fig. 1a). The ATP Assay is based on the detection of adenosine triphosphate (ATP) by the luciferin-luciferase reaction, the amount of ATP reflected by luminescence intensity is proportional to the number of viable cells. The ATP assay-based IC_50_ value was 3.7 µmol/L (95% CI [2.4, 5.2]; Fig. 1c, e).

Next, we investigated the cytotoxic effects in tumour spheroids. First, we performed the ATP assay, considered a reliable and reproducible method to determine the viability of large spheroids^38^. The ATP assay-based IC_50_ value for MCF-7 spheroids was 6.1 µmol/L (95% CI [5.5, 6.8]; Fig. 1d, f).

Taken together, ATP assays suggest higher cytotoxicity of PhO dendrimer in the 2D cell culture (3.7 µmol/L, 95% CI [2.4, 5.2]) than in the 3D spheroid culture (6.1 µmol/L, 95% CI [5.5, 6.8]).

Next, we compared the new PhO cytotoxicity data on MCF-7 cancer cells with our previously published results from non-cancer mammalian cell lines, namely B14 (*Cricetulus griseus*), BRL (*Rattus norvegicus*), NRK (*R. norvegicus*)^20^ , (Fig. 1 g). The IC_50_ value of PhO dendrimer in the MCF-7 cancer cell line (1.1 µmol/L, 95% CI [1.0, 1.3]; MTT assay) suggests its higher toxicity for cancer cells compared to mammalian non-cancer cells (B14 = 6.2 µmol/L, 95% CI [5.4, 7.0]; BRL = 7.8 µmol/L, 95% CI [6.0, 9.6]; NRK = 9.7 µmol/L, 95% CI [8.3, 11.1]; Fig. 1b). The IC_50_ values from the CV assay performed on mammalian non-cancer cell lines^20^ and ATP cell viability done here also suggest higher cytotoxicity of PhO towards the cancer cells compared to non-cancer cells; however, this comparison is limited by the difference between those two assays (see Discussion).

**Figure 2:**
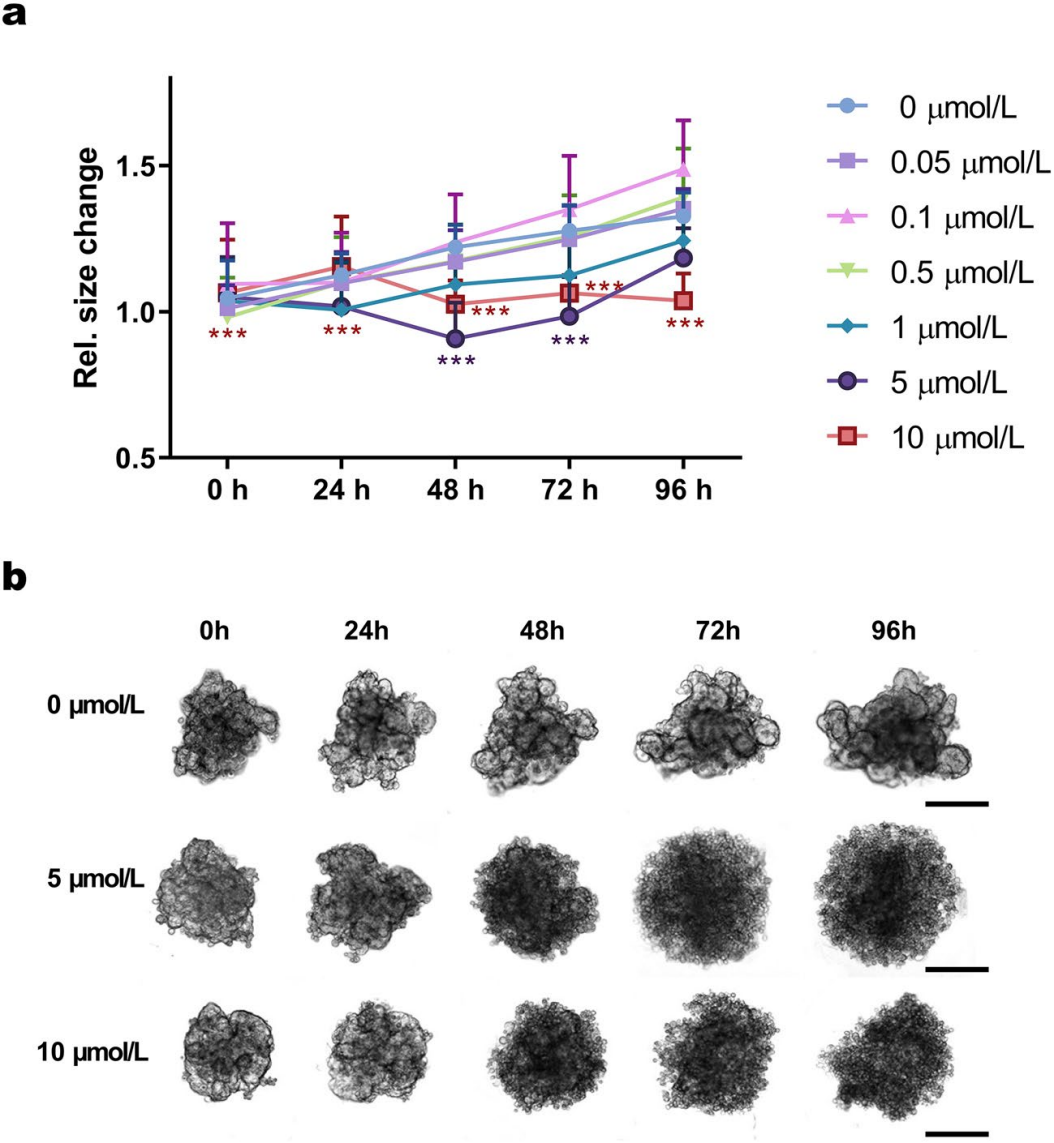
3D In vitro Assays. (**a**) Dendrimer cytotoxicity as represented by MCF-7 spheroid growth curves. The levels of significance: ***p < 0.001, between dendrimer concentrations and the control. Statistical significance was determined using the two-way ANOVA followed by the Dunnett’s post hoc test. (**b**) Changes in spheroid morphology after incubation with PhO. (-) Control spheroids incubated without PhO. Scale bars = 200 μm.

Another method to establish PhO dendrimer cytotoxicity was the Spheroid growth inhibition assay, in which the size and morphology of MCF-7 spheroids co-incubated with PhO (0.05, 0.1, 0.5, 1, 5, 10 µmol/L) were monitored over time using optical microscopy (Fig. 2a). This analysis revealed changes in size and compactness of spheroids co-incubated with the PhO dendrimer at 5 µmol/L and 10 µmol/L concentrations (Fig. 2b). PhO-induced effects were recognisable already at 48 h at both concentrations, leading to rapid spheroid disintegration (a more severe effect was observed at 10 µmol/L). The reduction in spheroid size and compactness at 10 µmol/L PhO was still observed after 96 h of incubation.

Both the ATP (Fig. 1d, f) and the spheroid growth inhibition assays (Fig. 2) indicated a concentration-dependent cytotoxicity of PhO dendrimer in all tested systems.

### Biodistribution of the PhO-Cy5 conjugate in spheroids

**Figure 3:**
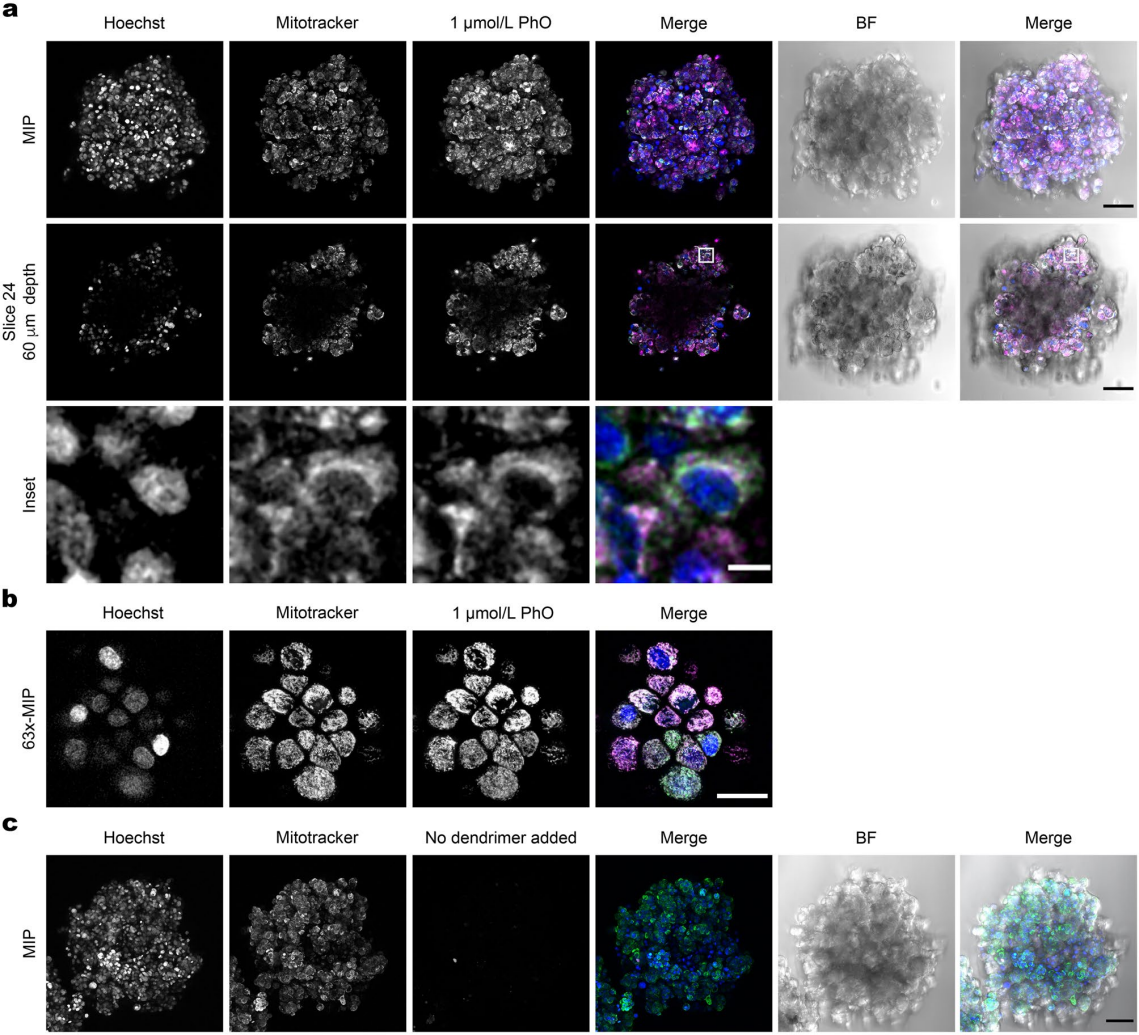
Biodistribution of the PhO-Cy5 Conjugate in Spheroids. (**a**) A maximum intensity projection (MIP) of a confocal stack of an MCF-7 spheroid cultivated for 72 h in the presence of 1 µmol/L PhO-Cy5. A single slice is shown with an inset highlighting the mitochondrial localization of PhO-Cy5. The images of individual channels of the shown slice were despeckled using Fiji and the insets were further adjusted using 0.75 σ Gaussian blur. Hoechst (blue), Mitotracker Red (green), and PhO-Cy5 (magenta). Black scale bar = 100 μm, white scale bar = 10 μm. (**b**) Another MCF-7 spheroid stained identically as in (**a**), imaged with an oil 63x/1.40 N.A. objective to improve the resolution for visualising mitochondrial localization. Scale bar = 25 μm. (**b**) MIP of a control MCF-7 spheroid cultivated without PhO-Cy5, stained and imaged identically as in (**a)**. BF—Bright Field.

To investigate PhO subcellular distribution, MCF-7 tumour spheroids were treated with the PhO-Cy5 conjugate, Hoechst 33342 to stain DNA, and Mitotracker red dye to stain mitochondria. Using confocal microscopy, we found a significant accumulation of PhO-Cy5 inside the spheroid, specifically in the mitochondrial compartment (Fig. 3).

### In vivo toxicity evaluation

**Figure 4:**
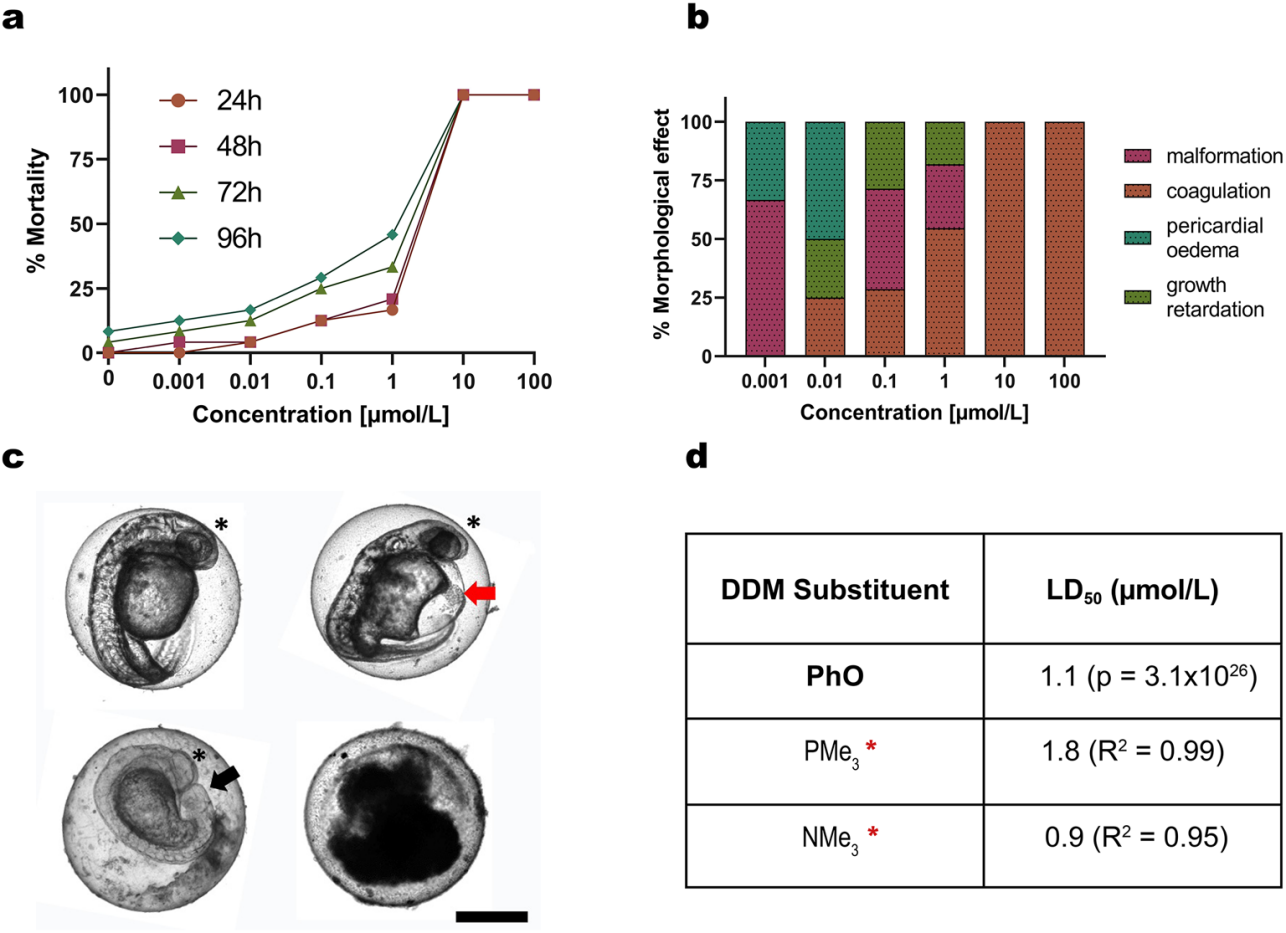
FET Results for Zebrafish Embryos Exposed to PhO. (**a**) Concentration–response curve calculated for the evaluated FET time points (24, 48, 72, 96 h). Note: The absence of error bars means a single FET experiment with 24 embryos per concentration counted as individual replicates. (**b**) Representation of the observed morphological effects in individuals showing lethal signs caused by exposure to the given concentration of PhO. (**c**) Illustrative micrographs of the toxicity endpoints in zebrafish embryos exposed to PhO. **1** Untreated embryo at 48 h post-fertilisation used as a negative control. **2** Embryo displaying yolk and heart oedema (red arrow). **3** Embryo growth retardation and failed tail detachment (black arrow). **4** Embryo coagulation. The head area is indicated by an asterisk. Scale bar = 200 µm. (**d**) Comparison of LD_50_ between PhO and previously published carbosilane dendrimers with—PMe_3_ (methyl phosphonium) and -NMe_3_ (methyl ammonium) substituents. Red asterisk indicates previous work by^39^. DDM—dendrimer.

To evaluate the PhO toxicity at the organismal level, we performed FET at PhO concentrations ranging from 0.001 to 100 µmol/L (Fig. 4).

The observed toxicity effects became more obvious with longer exposures to higher concentrations (Fig. 4a). PhO induced 100% mortality (by embryo coagulation) after 24 h of incubation at 10 and 100 µmol/L concentrations, suggesting that the higher concentrations of PhO disrupt the process of embryo gastrulation, leading to failed organogenesis. At concentrations 0.001—1 µmol/L, malformations (2/24 embryos at 0.001 µmol/L; 3/24 embryos at 0.1 µmol/L; 3/24 embryos at 1 µmol/L), growth retardation (1/24 embryos at 0.01 µmol/L; 2/24 embryos at 0.1 µmol/L; 2/24 embryos at 1 µmol/L), and pericardial oedema (1/24 embryos at 0.001 µmol/L; 2/24 embryos at 0.01 µmol/L) were recorded (Fig. 4c). These abnormalities can compromise the viability of the developing fish and indicate that even at lower concentrations, PhO can interfere with normal embryogenesis. The cumulative score of mortality for concentration was used to calculate LD_50_ 1.1 µmol/L (95% CI [0.7–1.5], *p* = 3 × 10^26^) after 96 h of incubation. When compared to the LD_50_ values obtained in previous studies on carbosilane dendrimers with -NMe_3_ (methyl phosphonium) and -PMe_3_ (methyl ammonium) substituents^39^, PhO showed comparable levels of in vivo toxicity (Fig. 4d).

### Biodistribution of PhO-Cy5 conjugate in zebrafish embryos

To gain insight into the biodistribution pattern of the PhO-Cy5 conjugate within a whole organism, zebrafish embryos were chosen as an appropriate model due to their transparent nature and ease of manipulation. The distribution of PhO-Cy5 after co-incubation with intact or dechorionated embryos shows that PhO-Cy5 partially adheres to the chorion and to the body surface of dechorionated (hatched) embryos with an apparent single accumulation site in the olfactory epithelium in the olfactory pits (Fig. S1; Supplement). There was no significant accumulation of PhO-Cy5 in the internal organs detected, indicating a minimal tendency for dermal absorption of PhO-Cy5.

**Figure 5:**
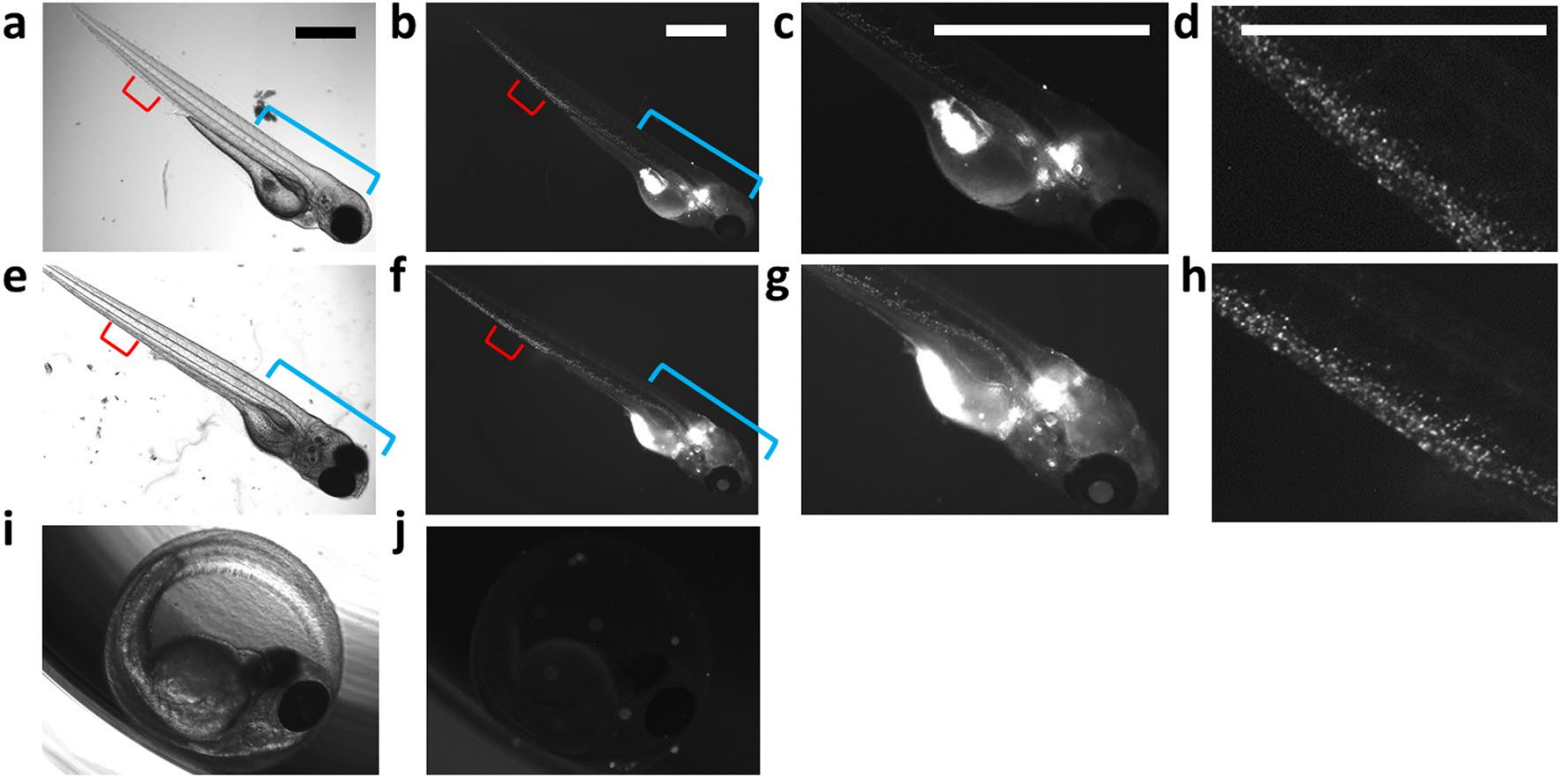
Biodistribution of PhO-Cy5 Conjugate after Injection into Zebrafish Embryos. (**a**–**d**) Biodistribution 24 h postinjection. (**e**–**h**) Biodistribution 96 h postinjection. (**a**, **e**) Bright field image; (**b**, **f**) PhO-Cy5 fluorescent signal detected within the yolk sack, gastrointestinal tract, and vascular system. (**c**, **g**) Detail of the frontal part of embryo (corresponding to the blue line in the whole-body image). (**d**, **h**) Detail of the tail region (corresponding to the red line in the whole-body image). (**i**, **j)** Negative control. Scale bars = 200 µm.

The biodistribution and accumulation of PhO-Cy5 using direct administration were analysed at 24 and 96 h post injection (48 h and 120 h post fertilisation, respectively; the organ systems are mostly developed at 54 h post fertilisation) (Fig. 5). The injection of PhO-Cy5 conjugate into the yolk sac of 24-h post-fertilisation embryos resulted in a notable biodistribution pattern. Primarily, accumulation was observed in the gastrointestinal system, specifically the yolk sack extension and the developing stomach, liver, and intestine. This pattern suggests a direct route of transport from the yolk sac to these organs, likely facilitated by the inherent physiological connections and the digestive process. In addition to the gastrointestinal tract, the PhO-Cy5 conjugate was detected in the vascular system of the embryos, specifically within the common caudal vein and the posterior caudal vein, which became more evident at a later stage (96 h postinjection; Fig. 5h). This finding was consistent with our expectations, as these major blood vessels can facilitate systemic dissemination of the nanoparticles throughout the organism. The absence of fluorescence signals in brain tissues implies that PhO-Cy5 is not capable of penetrating the blood–brain barrier (established at approximately 72 h postfertilisation).

## Discussion

In the present study, we evaluated in vitro and in vivo toxicity and biodistribution of a novel methoxyphenyl phosphonium carbosilane dendrimer (PhO), a possible mitochondria-targeting vector for cancer therapeutics.

Important to our research was the incorporation of 3D tumour spheroids, as they more closely mimic the complexity and architecture of in vivo tumours^40^. In addition, the spheroids more accurately simulate drug resistance^41^, allowing for a more realistic evaluation of the behaviour of the PhO dendrimer in a tumour environment. The dendrimer exhibited slightly higher cytotoxicity in the monolayer 2D cancer cell cultures than in 3D spheroid cultures (Fig. 1c, d), as revealed by the ATP assay IC_50_ values. This difference could be due to the more complex, multicellular structure of 3D spheroids, which limits the penetration of the dendrimer. This difference in cytotoxicity between 2 and 3D models underscores the importance of incorporating more physiologically relevant 3D tumour models to gain a more accurate understanding of dendrimer activity in a tumour context.

When comparing our ATP assay results to previous studies that utilised CV assays^20^, we noted an apparent difference in the IC_50_ values for PhO in cancer and non-cancer cells (Fig. 1g). Specifically, the CV-based IC_50_ values were higher, indicating a lower cytotoxicity of PhO to non-cancer cells compared to the IC_50_ from the ATP assay performed on cancer cells. Generally, the differences in the IC_50_ values between the CV and ATP assays might arise from the fundamental differences in what each assay measures, which affects their sensitivity to the cytotoxic effects of PhO. The CV assay measures cell viability by staining viable cells that are adherent to the plate, while the ATP assay measures cell viability based on the presence of ATP, which is a marker of metabolically active cells. The CV assay, therefore, might not be as sensitive as the ATP assay to changes in cell metabolic activity, such as those induced by PhO in cancer cells. In contrast, the ATP assay can detect reductions in cell metabolic activity even before cell death occurs, which could explain the higher sensitivity of the ATP assay to the cytotoxic effects of PhO in cancer cells. This difference underscores the importance of selecting the appropriate cell viability assay depending on the specific mechanism of action of the compound under investigation. For a compound like PhO that might target cell metabolism^20^, an ATP-based assay might provide a more sensitive measure of its cytotoxic effects. Alternatively, the difference might be attributed to the inter-species differences among human and other mammalian cell types. Further studies are needed to expand our findings and fully elucidate the differential cytotoxicity of PhO in cancer and non-cancer cells. Overall, the IC_50_ values obtained from each assay and model provide early indications of cytotoxicity of PhO in the low micromolar range in both cancer and non-cancer cells.

Organelle-specific targeting of bioactive molecules has received much attention recently^42^, mainly due to a maximal therapeutic effect with a minimum of side effects^43^. Interestingly, we found that the PhO dendrimer is specifically targeted to the mitochondria. The mitochondrial localization of PhO is consistent with earlier predictions based on our 2D culture assays^20^. The cationic and lipophilic nature of PhO dendrimer^21^ is essential for any mitochondria-targeting molecule^44^. MTT assay IC_50_ values suggested a higher toxicity of the dendrimer for the mitochondria of cancer cells compared to non-cancer cells (Fig. 1a, b). This could be attributed to the different metabolic characteristics of cancer cells, leading to higher mitochondrial activity^45^.

Consequently, the mitochondria-targeting PhO dendrimer may, by itself, cause increased cytotoxic effects in cancer cells. This mitochondrial specificity of the dendrimer could potentially disrupt key mitochondrial functions in cancer cells, inducing cell death^46^. Moreover, its selective cytotoxicity could minimise off-target effects on non-cancerous cells, enhancing its therapeutic profile. Specific mitochondrial mechanisms affected by PhO in cancer cells warrant further investigation.

While the cellular biodistribution of nanoparticles provides valuable insight into compartment accumulation, it is equally important to investigate their in vivo fate. In vivo biodistribution studies provide information on the level of the entire organism, such as how nanoparticles are eliminated from circulation, which organs they accumulate in, and potential systemic toxic effects. This data is essential for determining the safety and effectiveness of nanoparticles and optimising their design for specific applications.

To complement our in vitro findings, we evaluated the biodistribution and toxicity of the PhO dendrimer in vivo using the zebrafish model, which is an ideal tool for rapid assessment of nanomaterials^47^. The biodistribution pattern observed after direct yolk sac injection of PhO-Cy5 in zebrafish embryos provided insight into the nanoparticle transport mechanisms in this model. The primary accumulation in the gastrointestinal tract (yolk sack, the developing stomach, liver, and intestine) indicates that PhO-Cy5 is directly transported from the yolk to these connected digestive organs. This is likely facilitated by the physiological links between these structures and the developmental processes occurring during the stages examined. Detection within the common caudal vein and posterior caudal vein demonstrates that PhO-Cy5 can also enter circulation and distribute systemically after yolk sac administration. The increasing vascular fluorescence at later time points supports this. However, the lack of brain accumulation signifies that PhO-Cy5 probably cannot cross the blood–brain barrier, which is established 72 h postfertilisation in zebrafish. Overall, these findings suggest that yolk sac injection enables both gastrointestinal and vascular transport of PhO-Cy5 in developing zebrafish.

The PhO-Cy5 conjugate is not degraded or eliminated from the tissues, which should be considered in potential clinical applications since the accumulation of nanoparticles was shown to upregulate the immunological response in zebrafish embryos (characterised by the presence of neutrophils and macrophages around the particles^48^). The accumulation of nanoparticles in the tissues can lead to an increase in the immune system’s activity, which can have both beneficial and harmful effects. On the one hand, it can help eliminate the foreign particles and prevent potential toxicity. On the other hand, it could lead to chronic inflammation and tissue damage, which can negatively impact the efficacy and safety of nanomedicines. The extent of the immunological response depends on several factors, such as the size, shape, surface charge, and surface coating of the nanoparticles, as well as the route and duration of exposure^49^. Moreover, the immunological response can vary among different species and individuals, which makes it challenging to predict the clinical outcomes of nanoparticle-based therapies based solely on non-mammal in vivo studies.

While our study utilises the widely accepted method of using fluorescently labelled nanoparticles in both in vitro and in vivo biodistribution studies^50–52^, it is important to note the potential limitations of this technique. Specifically, fluorescent tags may affect the distribution of nanoparticles, obscuring our understanding of their true behaviour^53^. Consequently, alternative labelling techniques, such as radioisotopes or magnetic resonance imaging, should be utilised to provide independent confirmation of nanoparticle biodistribution^54^.

The FET results indicate that PhO exposure during early development has detrimental impacts on zebrafish embryos in a concentration-dependent manner. The coagulation at the highest concentrations of 10–100 μmol/L PhO suggests these levels cause systemic toxicity that disrupts critical developmental processes like gastrulation. This leads to the failure of proper organ formation and embryo death. At lower concentrations (0.001–0.1 μmol/L), the observation of malformations, slowed growth, and pericardial oedema indicates PhO can interfere with specific morphogenic events during embryogenesis even at sublethal levels.

The specific abnormalities seen can provide clues into which developmental signalling pathways may be disrupted by PhO toxicity. Pericardial oedema suggests impairment of cardiac development; growth retardation and malformations indicate broader developmental delay, which could stem from interference with developmental morphogen pathways. Given the conserved nature of developmental signalling across vertebrates, additional research should be conducted to understand the molecular and cellular impacts of low, non-lethal levels of PhO exposure that lead to the developmental abnormalities seen in zebrafish embryos and provide broader insights into how PhO may disrupt development in other vertebrates, including humans.

The LD_50_ values from our FET study fit within those reported for other carbosilane dendrimers^39^, suggesting a similar level of in vivo toxicity as in dendrimers with -NMe_3_ and -PMe_3_ substituents, which were proposed to be suitable candidates for clinical applications. These LD_50_ values serve best as a comparative metric for toxicity between various dendrimers or nanoparticles with potential as cancer therapeutics. However, it’s crucial to recognise that the translation of these values to potential patient exposure levels is a complex process that demands careful consideration of a multitude of factors. Thus, while LD_50_ values offer a comparative framework, further investigations should employ more physiologically relevant models, such as mammalian models. Once proven safe in mammalian model animals, human pharmacokinetic studies and dose-finding clinical trials would be needed to determine the optimal, patient-specific exposure levels.

Our study has several limitations that need to be taken into consideration. The 3D spheroid models, while valuable, lack the full complexity of in vivo tumours (lacking critical components like immune cells and vasculature). The use of a single cancer cell line may limit the generalizability of our results due to varying metabolic characteristics across different cancer types. The zebrafish model, while effective for initial assessments, may not fully reflect dendrimer behaviour in mammalian systems. Also, the use of fluorescent tags could potentially alter dendrimer behaviour and distribution.

While our study provides a good starting point, further studies on the pharmacokinetics and efficacy of the therapeutic payload delivery of PhO dendrimers are needed to determine optimal conditions for studies in mammals.

## Conclusions

In summary, our results indicate that the novel methoxyphenyl phosphonium carbosilane dendrimer (PhO) might exhibit selective cytotoxicity towards cancer cells compared to non-cancer cells. The 3D spheroid experiments revealed slightly lower cytotoxicity compared to 2D cultures, highlighting the importance of physiologically relevant models. Using confocal microscopy, we demonstrated the specific mitochondrial accumulation of PhO in tumour spheroids, consistent with its cationic, lipophilic nature. The zebrafish embryo experiments revealed biodistribution primarily in the gastrointestinal and vascular systems after yolk sac injection without crossing the blood–brain barrier. Compared to related -NMe_3_ and -PMe_3_ carbosilane dendrimers, PhO displayed a similar in vivo toxicity profile based on LD_50_ values in the fish embryo test.

While further studies are needed to confirm our findings, particularly in mammalian models, our results provide strong preliminary evidence for the promise of PhO as a potential mitochondria-targeting vector for cancer cell delivery. The next key steps should include investigating the specific mitochondrial mechanisms of toxicity in cancer cells, pharmacokinetics and therapeutic payload delivery using PhO in mammalian models, and confirming biodistribution patterns with alternative labelling techniques. With careful optimisation, PhO dendrimers hold potential as vectors for targeted cancer therapeutics with improved selectivity.

### Supplementary Information

Supplementary Information.

## Data Availability

The data that support the findings of this study are available from the corresponding author, [ML], upon request.
