# Supplementary material for: Biodistribution and toxicity assessment of methoxyphenyl phosphonium carbosilane dendrimers in 2D and 3D cell cultures of human cancer cells and zebrafish embryos
Source: Sci Rep. 2023 Sep 19;13:15477. doi: 10.1038/s41598-023-42850-3 (PMC10509138; doi:10.1038/s41598-023-42850-3)
Supplement: Supplementary file 1 — Supplementary Information. [file 41598_2023_42850_MOESM1_ESM.docx]

**Supplemental Information**

**Biodistribution and Toxicity Assessment of Methoxyphenyl Phosphonium Carbosilane Dendrimers in 2D and 3D Cell Cultures of Human Cancer Cells and Zebrafish Embryos**

Zuzana Žmudová^1^, Zuzana Šanderová^1^, Michaela Liegertová^1^*, Stanislav Vinopal^1^, Regina Herma^1^, Luděk Sušický^1^, Monika Müllerová^1,2^ Tomáš Strašák^1,2^ and Jan Malý^1^

*^1^CENAB, Faculty of Science, Jan Evangelista Purkyně University in Ústí nad Labem, Czech Republic*

*^2^Institute of Chemical Process Fundamentals of the CAS, Prague, Czech Republic*

*Corresponding author


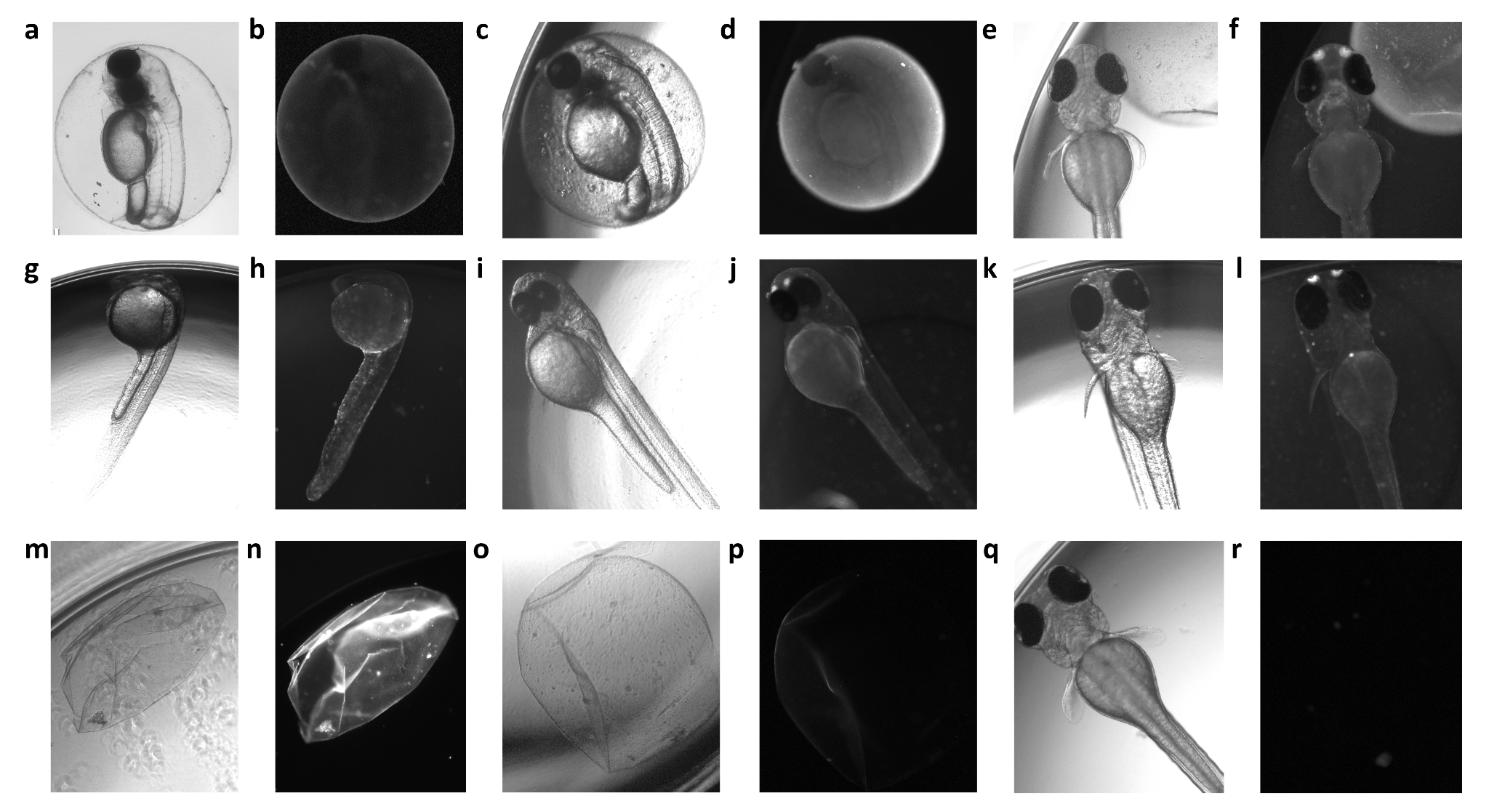
**FIGURE S1: Biodistribution of PhO-Cy5 conjugate after co-incubation with intact and dechorionated embryos**

**a, b** Biodistribution in intact embryos after 24 hours of co-incubation (**a** in bright field, **b** in fluorescent mode). **c, d** after 48 hours of co-incubation. **e, f** after 72 hours of co-incubation (embryo hatched). **g, h** Dechorionated embryos after 24 hours of co-incubation. **i, j** after 48 hours of co-incubation. **k, l** after 72 hours of co-incubation (as compared to the negative control **q, r**). **m, n** detail of the chorion after 72 hours co-incubation as compared to negative control (**o, p)**
